# Visualizing the Impact of Art: An Update and Comparison of Current Psychological Models of Art Experience

**DOI:** 10.3389/fnhum.2016.00160

**Published:** 2016-04-26

**Authors:** Matthew Pelowski, Patrick S. Markey, Jon O. Lauring, Helmut Leder

**Affiliations:** ^1^Department of Basic Research and Research Methods, Faculty of Psychology, University of ViennaVienna, Austria; ^2^BRAINlab, Department of Neuroscience and Pharmacology, University of CopenhagenCopenhagen, Denmark

**Keywords:** art, cognitive models, information processing, emotion, evaluation, aesthetic experience

## Abstract

The last decade has witnessed a renaissance of empirical and psychological approaches to art study, especially regarding cognitive models of art processing experience. This new emphasis on modeling has often become the basis for our theoretical understanding of human interaction with art. Models also often define areas of focus and hypotheses for new empirical research, and are increasingly important for connecting psychological theory to discussions of the brain. However, models are often made by different researchers, with quite different emphases or visual styles. Inputs and psychological outcomes may be differently considered, or can be under-reported with regards to key functional components. Thus, we may lose the major theoretical improvements and ability for comparison that can be had with models. To begin addressing this, this paper presents a theoretical assessment, comparison, and new articulation of a selection of key contemporary cognitive or information-processing-based approaches detailing the mechanisms underlying the viewing of art. We review six major models in contemporary psychological aesthetics. We in turn present redesigns of these models using a unified visual form, in some cases making additions or creating new models where none had previously existed. We also frame these approaches in respect to their targeted outputs (e.g., emotion, appraisal, physiological reaction) and their strengths within a more general framework of early, intermediate, and later processing stages. This is used as a basis for general comparison and discussion of implications and future directions for modeling, and for theoretically understanding our engagement with visual art.

## Introduction

Today, millions of individuals across the globe regularly encounter works of art. Whether, in the museum, the city-center, or on the web, art is an omnipresent part of human life. Underlying the fascination with art is a uniquely impactful experience. When individuals describe noteworthy art or explain why they go to museums, most often they refer to a complex mix of psychological events (Pelowski and Akiba, 2011). Art viewing engenders myriad emotions, evokes evaluations, physiological reactions, and in some cases can mark or alter lives. Reactions can also differ greatly between individuals and settings, or evolve within individual experiences themselves.

Understanding this multifaceted impact of art is key for numerous areas of scholarship—including all humanities, sociology, evolution, museum education, art history—and is especially key for psychology and empirical art research (Leder, 2013). The relevance of the topic has only grown in the past decade, which has seen a burgeoning of psychological aesthetics through the emergence of new empirical methods, growing interest in affect and emotion, and new integration between behavioral and neurophysiological analyses.

Perhaps most important, recent approaches have been accompanied by attempts to model the underlying processes of art engagement (Leder, 2013). These models build from recent trends in cognitive science, employing a visual approach for highlighting the interconnections and outcomes in our experience. They posit key inputs, and connect these via a flow of processing stages (often utilizing a box-and-arrow design) to outcomes or psychological implications. Thus, by offering a process-driven articulation of psychological elements, models have become the indispensable basis for shaping hypotheses. Even more, by stepping beyond written theory and articulating ideas within a visual frame, models can emphasize processes and important elements that previously might have been merely implicit. Thus, the visual models themselves often become the working theories for art study, and determine empirical research.

However, current modeling also suffers from several limitations, which hamper our ability to fully compare and understand approaches. Models are often made with different emphases and visual grammars. There are often also different arrangements of processing stages or focus on different portions of the processing sequence. Psychological inputs and outcomes are also often differently considered, or can be omitted from the processing sequence. Thus, we often lose the major theoretical benefit—a clear connection between inputs, processes, and outputs—that can be had from placing ideas into a visual form. It is also difficult to consider various models' overlaps or major differences when explaining specific reactions to art, and thus difficult to articulate how they might contribute to our understanding of art experience.

This is the goal of this paper, which represents our attempt to provide a comparison of current key modeling approaches, and involving their translation into a comparable visual format. We do this by reviewing six influential approaches to art experience, as well as supporting literature by the same authors, and place these into a model form. For existing models, we adapt the previous approaches to a unified layout, and also suggest additions or changes based on our literature review. When an author's idea does not yet have a visual form, we newly create models based on their arguments. Through our review, we also give specific consideration to outputs or psychological implications for art experience, as well as general organization around early, intermediate and late processing stages. We end with a synthesis and discussion of avenues for future research. In this review, we have chosen approaches, which, we feel, have come to be bases for the past decade of general empirical art-viewing research, and which employ a cognitive or information processing focus. Although this paper can, admittedly, only address a small selection of models, by providing this analysis, we hope to create one more useful tool for advancing understanding of art processing and modeling research.

## Review: key model components and previous approaches to modeling art

Before beginning, it is instructive to briefly review what aspects *should* be considered in models of viewing art, and which will provide the material for this paper's comparison. Psychological models generally have three main components. These include: (1) inputs that feed into experience. These might include personality of the viewer, social or cultural setting, background affective state, other context (e.g., Jacobsen, 2006), as well as the specific artwork body and its history (Bullot and Reber, 2013); (2) processing mechanisms, which act on the inputs in specific stages (explained further below); and (3) mental and behavioral consequences (outputs) that arise from processing art. While it is the second stage of actual processing that makes up the bulk of models we will review, it is these outputs that constitute their implicit goal of addressing art interaction, and also the frame for this paper's review.

A literature review suggests multiple output examples. We have given these short labels, which will be used in the following discussions, and which can be divided into four main clusters: First, art has the capability to influence basic aspects of affect or the body. This can come from: (1) *Affect*, specific emotions/moods evoked by content or derived from the act of viewing; (2) *Physiology*, such as heart rate, skin conductivity, or other processes of the autonomic nervous system (e.g., Tschacher et al., 2012); and (3) *Actions*, for example gesture, eye movement, or physical movement during art reception.

Art also has been connected to numerous aspects of perception and understanding (e.g., see Leder et al., 2004), including: (4) *Appraisals* or particular judgments (beauty, liking); (5) *Meaning-making* as well as ability to strengthen conceptions, help us to learn, challenge our ideas, or even lead to insight. (6) *Novelty:* Art can impact what we see, induce changes in visual or perceptual experience involving new attention to physical aspects.

There are also elements which are more art-specific, or which are particularly salient in reports of art experience: (7) *Transcendence*: feelings of more sudden change, epiphany, or catharsis (Pelowski and Akiba, 2011); (8) *Aesthetic* mode: “aesthetic” emotions and responses, which might involve a state of being, whereby one detaches or uncouples from concerns or everyday life perceptions, often related to periods of contemplation or harmonious enjoyment, as well as potential positive reaction to negatively-valenced or troubling art (Cupchik et al., 2009). (9) *Negative* affect: Art can also evoke negative reactions such as disgust, queasiness or anger—outcomes that particularly require an explanation in models of experience (Silvia, 2009).

Art is also argued to create longitudinal impacts. These include: (10) *Self-adjustment*, changes in one's personality, worldview, cognitive ability (Lasher et al., 1983), or in the relation between art and viewer. This might also include a deepened ability to appreciate art or a more general improvement in visual-spatial ability (Funch et al., 2012). (11) *Social*: Art also may guide social behavior—e.g., in rituals or institutions—or lead to social ends such as indoctrination or social cohesion (Dissayanake, 2008). (12) *Health*: art may even have general impact on health and wellbeing, for example through reduced stress (Cuypers et al., 2012).

### A brief note on previous art modeling research

The above aspects have been the main focus for attempts to explain interaction with art. Models—as a result of systematic, scientific endeavor—can be traced back to at least the work of Berlyne (e.g., Berlyne, 1960, 1974; see also Funch, 2013 for review), who revived focus on art within empirical aesthetics, integrating a psychophysiological and cognitive perspective. Looking to physiological arousal, he posited opposing reward and aversion systems tied to “collative” art properties. He was followed by Kreitler and Kreitler (1972), who took a largely cognitive and Gestalt approach, arguing that artwork content and structure make it a carrier of multiple meanings that can stimulate understanding and emotion. Similarly, based on Gestalt perception, (e.g., Arnheim, 1966) considered the means whereby structural unity of artworks (balance, grouping) and individual features drive responses. This was followed by, for example, Martindale (Martindale, 1988; Martindale et al., 1988), who more fully emphasized cognition, focusing on matching of schema and stimulus, and proposing prototypicality as a key determinant for positive appraisal/affective response. These were followed by an even greater expansion of approaches. Notable examples include: Lasher et al. (1983), who proposed a cognition-based model of profound experience or insight; Ramachandran and Hirstein (1999), who gave one of the first attempts to posit universal rules for reactions and their underlying biological or neurological connections; and Jacobsen (2006) as well as Solso (1994), Vitz (1988), Zeki and Nash (1999), who presented an integrative neuro-cognitive theory. Other important approaches, many of which deal with specific aspects of viewing, also include: the fluency-based theory of aesthetic pleasure by Reber et al. (2004); Graf and Landwehr's (2015) updated consideration of fluency and visual interest; Van de Cruys and Wagemans (2011) account of rewarding reactions; Armstrong and Detweiler-Bedell's (2008) work with beauty; Funch's (2007) phenomenological model of art experience; Carbon (2011); Hekkert's (2012) design-based model; Bullot and Reber's (2013) integrated model of low-level processes and top-down integration regarding viewer knowledge of artwork history; and Tinio's (2013) consideration of creating/viewing art.

These approaches, among many others, give a basis for present modeling, notably pointing out the importance of individual elements such as beauty, pleasure. They also represent a research trend from emphasizing single, often simple visual elements to a more complex interplay of factors, which may drive emotion and physiological response. Especially cognitive approaches have also strongly contributed to the basic input-process-output form of the models we consider below.

## Current models and cohesive theories of interacting with art

What follows is a review of six models, which we feel, offer a good overview of present approaches to general empirical exploration of art experience. These again are not the only important models, as witnessed from the review above, but were chosen because they offer psychological explanations which are explicit in respect to underlying cognitive processes, and which are presently used in empirical consideration of outputs/inputs when viewing art. The following paragraphs will follow a repeated pattern: First, the background and main elements of each model are presented and put into a unified visual form. When a visual example has been previously produced by the models' authors, we have attempted to reproduce in verbatim the original structure and wording, with only some shifting in the location of elements. At the same time, we have taken the liberty of creating new models or new processing elements when this was deemed to be necessary. To distinguish from our own additions, previously created model components are shown in black, while our contributions are shown in blue. All models will thus have a standardized format, employing five components. Inputs and contextual factors are shown with rounded edges and depicted on the far left, processing stages in the middle, and outputs on the far right (distinguished by a gray band).

The middle section also incorporates a timeline (bottom), showing general ordering and designating early, intermediate, and late processing stages. These were included because the specific placement of components within these stages may be key in hypothesis-making, and the relative emphasis also varies greatly between the reviewed models. Although there is as of yet no agreed-upon distinction, generally the early stage refers to immediate, automatic, bottom-up visual processing and attention, while intermediate refers to more specific processes involving object recognition, classification and memory contribution. The late stage refers to more overt cognitive components such as reflection, association, or changes in viewer approach (Leder and Nadal, 2014). Thus, this factor provided one more point for comparison and for the ordering of model presentation below. In the time line, we also include designation of automatic or more overtly conscious processing, as this is mentioned in many approaches. Finally, specific outputs, using the above labels (see also **Table 2**), are placed in red circles at their suggested model location. If an output could be posited, yet was not explicitly considered by the authors, it is shown in a lighter shade.

### Chatterjee: neurological/cognitive model

We begin with the earliest model from the present group, and one which emphasizes early processing stages. This also makes a nice example of the present box and arrow design. This model was introduced by Chatterjee (2004, 2009, 2010; see Cela-Conde et al., 2011 for review), and has become a central tool for framing empirical assessments. It was designed to address cognitive and neuropsychological aspects, connecting processing stages to brain functioning. Chatterjee (2010) argues that visual interaction with art has multiple components and that experience emerges from a combination of responses to these elements. It draws its main theoretical emphasis from vision research (Chatterjee, 2004, 2010). Thus, it focuses primarily on three stages which are argued to correspond to the rough functional division of “early,” “intermediate,” and “late” human visual recognition (e.g., Marr, 1982).

**Figure 1:**
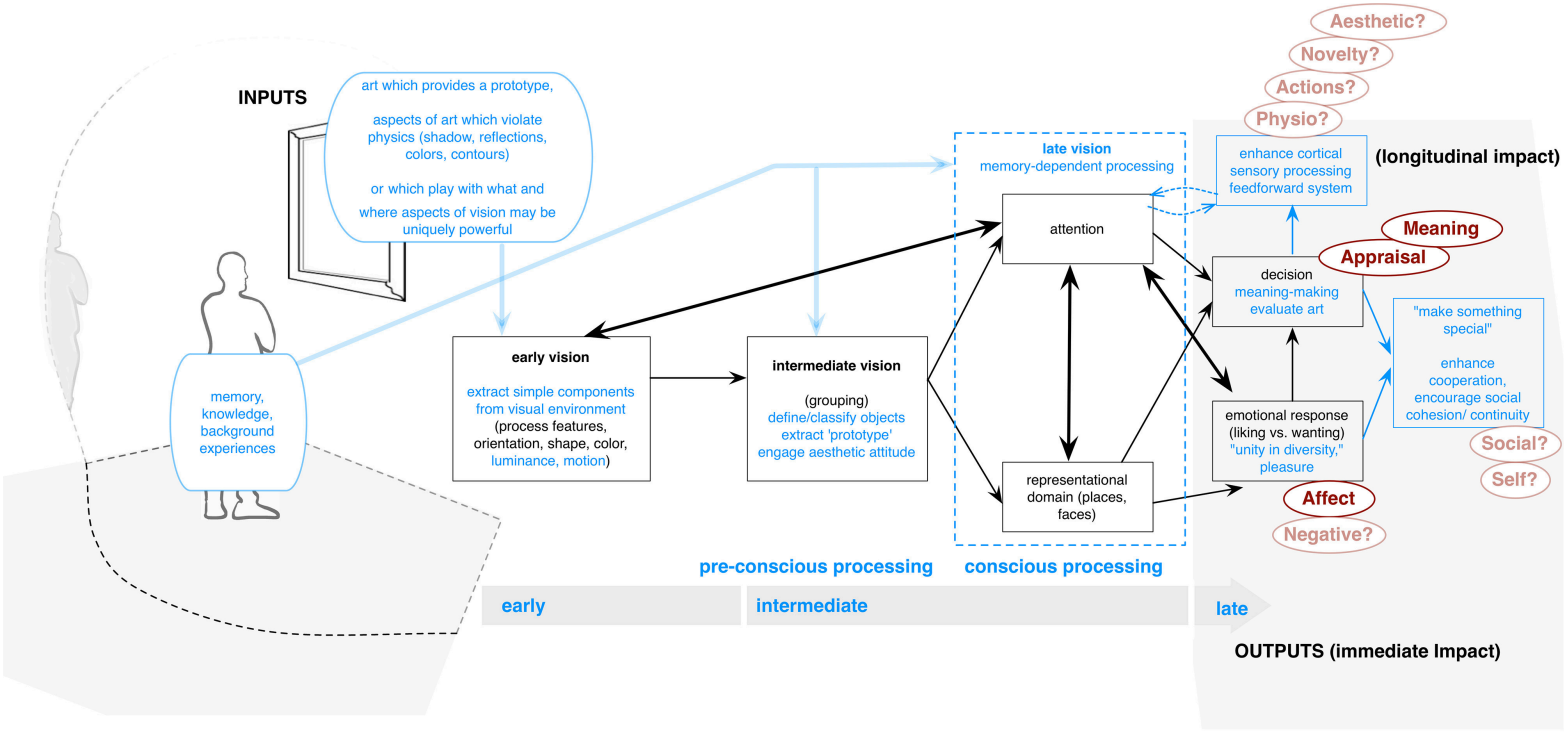
**Chatterjee model adapted from original visual model in Chatterjee (2004)**. Original elements shown in black. Additions not originally included in model shown in blue. If possible, original wording has been retained or adapted from model author's publications.

As shown in Figure 1, where we have reproduced the original model, Chatterjee posits that visual attributes of art are first processed, like any other stimulus, by extracting simple components (location, color, shape, luminance, motion) from the visual environment, and processing these in different brain regions^1^. Early features are subsequently either segregated or, most often, grouped to form larger units in intermediate vision. Here, elements help to define the object and to “process and make sense of what would otherwise be a chaotic and overwhelming” array of information (Chatterjee, 2004, p. 55). Late vision then involves selecting regions to scrutinize or to give attention, as well as evoking memories, attaching meaning, and assessing foci of specific evolutionary importance (e.g., faces, landscapes)^2^. Following recognition and assessment, evaluations are then evoked as well as emotions.

This model also provides an important basis for empirically approaching the role of the brain, with imaging studies having identified regions tied to its posited stages (Nadal et al., 2008). It affords a basis for making observations about how we progress in viewing, and how particular aspects—in relation to the way they are processed by the brain—impact judgments. For example, it suggests that one first perceives formal elements due to their importance in early and intermediate vision, while content is typically assessed in later vision (Chatterjee, 2010). Further, the model affords nuanced understanding of how processes may integrate. For example, the process of taking initially diverse perceptions from the first stage and grouping them within the second may explain satisfaction or interest often generated by complex art (Chatterjee, 2004), suggesting a “unity in diversity,” which itself is a central idea in aesthetics.

The model also highlights the transition from automatic to self-aware assessment. It is argued that the initial perception of many formal features (e.g., attractiveness, beauty), as well as intermediate grouping, occurs automatically (Chatterjee, 2010). This is followed by memory-dependent processing, where the perceiver's knowledge and background experiences are activated, and consequently, objects are identified, leading to experience-defining outcomes that result from often effortful and focused cognition, such as meaning-making and aesthetic judgments (Tinio, 2013). Thus, a general progression from bottom-up to top-down processing, and from low-level features to more complex higher-order assessments of art, is illustrated. This approach does not imply a strictly linear “sequence” (Chatterjee, 2004). Rather, processes may often run in parallel, and the individual may revisit or jump between stages (Nadal et al., 2008).

#### Model outputs

This model affords opportunity for discussion of several impacts from art (Zaidel et al., 2013 for similar review). First, the model first focuses on reward value, which is connected to numerous brain regions and specifically associated with the generation of pleasant feelings in anticipation and response to art (“**Affect**” in Figure 1)^3^. High-level top-down processes are also involved in forming evaluative judgments and thus represent another vital component of aesthetic experiences (**Appraisal**)^4^. The model proposes a fluency or mastery-based assessment, where success in processing leads to positive responses. Due to its tie to brain function in reward and pleasure areas, the model could potentially also account for **Negative** responses here, which would presumably be linked to failing to place and group visual aspects, although this had not been described (we have made this addition in the Figure). The processing of objects, extraction of prototypes, connection to memory and final decision would also presumably connect to meaning-making or understanding (**Meaning**). Zaidel et al. (2013, p. 104) also note that “neuroimaging studies have identified an enhancement of cortical sensory processing”^5^ during aesthetic experiences. This would involve attention and may be tied to physical **Action** (eye movements), **Physiology** (relating to enhanced brain activity in certain regions), and changes in perception (enabling perception of new aspects or **Novelty**). This process may also include self-awareness, monitoring of one's affective state or conflict resolution, which may play a role in bringing about final aesthetic emotion and judgment.

The above three aspects are also connected to the possibility for profound/**Aesthetic** experience. If intermediate processing involves perceptions of specifically compelling or pleasing qualities of an object (e.g., symmetry, balance, as well as content) these qualities are argued to engage frontal-parietal attention circuits. These networks may continue to modulate processing, as an individual continues looking, within the ventral visual stream. Thus, “a feed forward system,” as might be seen in the arrow connecting early vision to attention, is established “in which the attributes of an aesthetic object engage attention, and attention further enhances the processing of these attributes” (Chatterjee, 2004, p. 55), leading to heightened engagement and pleasure. This outcome may also be particularly unique for defining aesthetic experiences (Nadal et al., 2008). We have suggested this connection in our update to the model.

Chatterjee (2011) also suggests that the evolutionary or biological basis for human fascination with art may be tied to the interplay of three factors: (1) beauty, potentially linked to the evolutionary aspect of mate selection; (2) aesthetic attitude, or mental processes involved when apprehending objects, and which may connect with the idea of “prototypes” (presumably in early vision) which are preferred and may influence environmental navigation. Finally, (3) he notes cultural or socially-derived concepts of “making special” (e.g., Dissayanake, 2008), where ordinary objects are transformed by the artist and whereby the institutional frameworks that promote and display art may tie to adaptive importance in enhancing cooperation and continuity within human groups. This latter might then connect to more longitudinal impacts (**Social**).

#### Inputs

Regarding inputs (primarily the blue arrows from the model left side), Tinio (2013), in his review, notes that the intermediary stage of vision should involve processing that recruits access to memory and processing that involves higher-order cognitions such as the perceiver's knowledge and background experiences, which may also influence the final stage of meaning-making. Chatterjee (2004), referencing Ramachandran and Hirstein (1999), also notes the importance of several artwork qualities. He suggests that neural structures that evolved to respond to specific visual stimuli respond more vigorously to primitives. This may be both based on previous experience, while also explaining the specific power of abstract art. In later theoretical work, he explained how other design cues might further impact the viewer. For example, artists might play with certain art-processing elements—violating physics of shadows, reflections, colors, and contours—thereby engendering specific brain responses (Chatterjee, 2010). Artists' use of complex interactions between visual components within art may also create a specifically powerful response by causing interplay between the dorsal (“where”) and ventral (“what”) vision systems within the first and second stages. Because the dorsal stream is sensitive to luminance differences, motion, and spatial location, while the ventral stream is sensitive to simple form and color, their interaction may lead to a shimmering quality of water or the sun's glow on the horizon, as in impressionist paintings (see also Livingstone, 2002).

#### Suggested additions

Finally, in regards to possible additions, besides those discussed above, the model is heavily influenced by both beauty and visual research, as well as philosophical ideas of (e.g., Kantian) disinterest (Vartanian and Nadal, 2007). While the emphasis on detached reception and visual pleasure may adhere to classical (pre-modern) art examples, it does not touch many aspects of modern art experience. Notably this includes a more robust or differentiated explanation for emotions, and negative evaluations. While the integrated nature of the model does allow for discussion of what brain areas may be tied to changes in perception/emotion, there is no explanation of the driving force that may bring these about. This is especially clear in Chatterjee's (2011) discussion of the evolutionary role of art. As he explains, focusing on liking or other aesthetic judgment as our sole focus would be maladaptive. If “the most profound …experiences involve a refined liking, often described as awe or feeling the sublime, in which wanting has been tossed aside, […and where] individuals lose themselves in the experience,” the individual would be rendered “vulnerable”—“Entering an aesthetic attitude is dangerous.” However, this does not take into account adaptations in the viewer. Most notably, we would recommend adding longitude changes (see box on far right), relating to making special or **Social/Health**. We would also suggest adding an indication of the “feed forward loop,” noted in the theoretical writing, and which appears to be placed between *decision* and *attention* stages, as well as contextual aspects such as perceiver and art qualities.

### Locher et al.: early and intermediate visual processing

**Figure 2:**
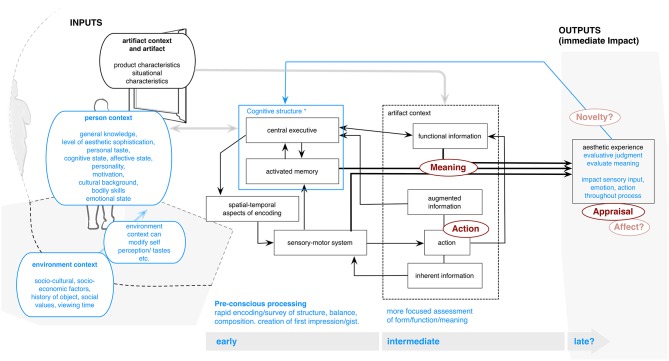
**Locher model (adapted from Locher, 1996; Locher et al., 2010)**.

Locher et al. (2007, 2010) also introduced a model, which deals with early/intermediate processing, primarily driven by empirical approaches to vision research. This model was conceived to describe the relationship between eye movements and scan patterns when processing visual art,^6^ and takes a somewhat different approach to the model layout, centering on three overlapping elements. The “person context” relates to both the personality inputs and the internal processes of the viewer. “Artifact context” refers to the physical aspects of the art. The “interaction space” details the physical meeting of viewer and art, mainly pertaining to eye movement and other outputs regarding actions. For the purpose of unification with the other approaches, we have moved these inputs to the model's left side and moved the processing stages to the middle (Figure 2).

The model involves two processing stages: First, similar to Chatterjee and following previous theory regarding vision (Marr, 1982; Rasche and Koch, 2002), Locher et al. argue that art viewing, like other perception, begins with a rapid survey of the global content of the pictorial field producing an initial “gist” impression (e.g., Locher, 2015) of global structural organization, composition and semantic meaning. This processing alone can activate memories, lead to emotion, and contribute to a first impression/evaluation. The detected gist information and resulting impression then drive the second stage, involving a more focused period of attention on form and functionality. This stage also involves focus on details or specific aspects of pictorial features in order to satisfy cognitive curiosity and to develop aesthetic appreciation. Information is gathered by moving the eyes over art in a sequence of rapid jumps, followed by fixations.

The authors posit that interaction in the second stage is also driven by the “Central Executive” (blue box inside “person context”)—consisting of “effortful control processes that direct voluntary attention” in a top-down, cognitively driven manner (Locher et al., 2010, p. 71). This also forms the “crucial interface” between perception, memory, attention, and action (depicted in the model's box labeled “spatio-temporal aspects of encoding”), and performs four important executive processes (following Baddeley, 2007): focusing, dividing, or switching attention, and providing a link between working and long-term memory. Thus there is argued to be a continuous, dynamic bottom-up/top-down interaction inside the Central Executive, involving assessed properties (form) and functionality of the object, and “viewer sensory-motor-perceptual” (i.e., visual) processes, as well as viewer cognitive structure. “Thus, as an aesthetic experience progresses, the artifact presents continually changing, ‘action driven’ affordances” (Locher et al., 2010, p. 71). These “influence the timing, rhythm, flow, and feel of the interaction.” Ultimately, “together the top-down and bottom-up component processes underlying thought and action create both meaning and aesthetic quality,” defining art experience. Like many of the other authors, Locher et al. note both automatic and more deliberate processing. Especially in the first phase, many aspects specific to a work—complexity, symmetry, organizational balance—are argued to be detected “automatically or pre-attentively by genetically determined, hard-wired” mechanisms (p. 73).

#### Outputs

The model especially involves focus on eye movements (**Action**). The authors' research—using eye tracking, as well as museum based observations and participant descriptions—specifically shows evidence for the initial gist processing, relating to a movement of the eyes over a large visual area and showing attention to elements perceived as compositional units (Locher et al., 2007; see also Locher and Nodine, 1987; Locher, 1996; Nodine and Krupinski, 2003). They also showed a later switch to focus on details as well as expressiveness and style/form elements. This also gives new evidence for discussion of **Appraisal** and **Meaning**-making, which can result from this sequential looking. They note that the stage of early processing can itself play an important role in these outcomes. Further, within their most recent model discussion, the authors classify three channels of information that one might create. These are composed of functional or conceptual information, inherent information (via affordances communicated in the object), and augmented information, presumably that which is changed or developed through viewing (**Novelty**). These outcomes would most likely come through directed looking in the second stage. The authors also note that emotion (**Affect**) may be evoked throughout the viewing process, however, they do not address how.

#### Inputs

The model notably argues for “two driving forces”: the “artifact itself” and a “person context that reflects the user's cognitive structures” (Locher et al., 2010, p. 72). With respect to the artwork, the authors (2010, p. 73) cite research (e.g., Creusen and Schoormans, 2005), which suggests “at least six ways” in which appearance influences evaluation and choice, including conveying aesthetic and symbolic value, providing quality impression, functional characteristics and ease of use, drawing attention via novelty, and communicating “ease of categorization.”

Regarding the viewer, the authors note that this input “contains several types of [acquired] information (semantic, episodic, and strategic),” and is also the “repository of one's personality, motivations, and emotional state,” all of which influence, in a top-down fashion, how viewers “perceive,” and “evaluate” (Locher et al., 2010, p. 73). They note that this might play a role in the second phase, where memory “spontaneously activates subsets of featural and semantic information in the user's knowledge base,” including the user's level of aesthetic sophistication, experience, tastes, education, culture, and personality. In addition, they note that “individuals are capable of rapidly detecting and categorizing learned properties of a stimulus,” for example characteristics of the artistic style and a composition's pleasantness and interestingness. “These responses occur by a rapid and direct match in activated memory between the structural features of an art object…and a viewer's knowledge” (p. 76). They also suggest that emotion itself may be an input. Locher (2015) also suggests that expertise of a viewer may play a particularly important role in the initial gist impression as well. For example, experts may give more importance to the initial impression and resulting affective reaction when appraising value or authenticity of artworks.

Locher et al. (2010) furthermore note importance of context. This includes social-cultural and socio-economic factors related to the object, its historical significance, symbolic associations and social value. These “contribute to a user's self-perception of his or her cultural taste” or aspirations (p. 78). These aspects are also argued to influence art interaction in a cognitively-driven, top-down fashion. However, the actual tie to outcomes is not discussed. Other mentioned factors include the environment, available time for viewing, and previous mood or exposures. For example, they cite studies in which individuals were primed by giving candy, resulting in better mood, which positively influenced evaluations, attention to details, and more balanced patterns of eye movement.

#### Additions

An integration of the discussion of action/eye-movement with emotion or evaluations would be useful. The authors also tend to place most aspects within the second stage and do not explain how possible sequences or patterns might lead to certain outputs *within* experience, nor how experience changes. This may also be a result of the lack of defined temporal flow in the model design. Interestingly, when Locher et al. (2007) gave individuals the opportunity to verbalize their initial gist reaction (roughly the first seven seconds), they did not find changes in the way individuals described artworks after this period, raising questions regarding the present delineation between stage one and two. They also do not consider longitudinal aspects. Better explanation of “augmented information”—one of the three channels of information argued to be created from looking in stage two—might give a point of entry for this. Further, in their empirical support for the model and its outputs, they note that their artworks were created by renowned artists “and are, therefore, presumably visually right” (Locher et al., 2007, p. 74). However, this raises the question of what makes a work “right,” and how less visually-successful art might be processed.

### Leder et al.: intermediate stages and aesthetic appreciation and judgments

A model that has its strengths in linking early and late processing, with focus on intermediate stages, is that of Leder and colleagues (Leder et al., 2004; updated in Leder, 2013; Leder and Nadal, 2014). This has also become perhaps the most prominent approach for empirical study (Vartanian and Nadal, 2007).

**Figure 3:**
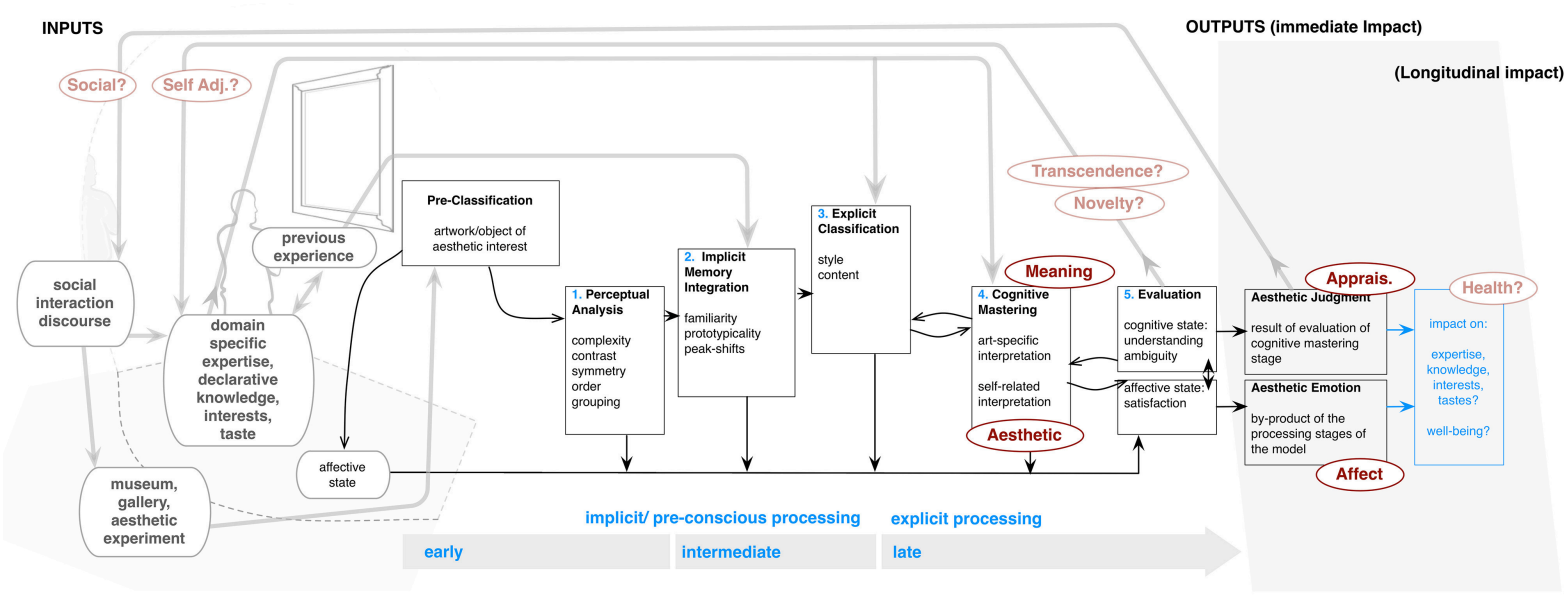
**Leder model (adapted from Leder et al., 2004; Leder and Nadal, 2014)**.

Based largely on the cognitive work of Kreitler, Kreitler, and Berlyne above (Leder et al., 2005), their model considers art experience as a series of information-processing stages, focusing largely on perceptual attunement to various formal factors in art. However, it also integrates this sensory information with “conceptual and abstracted” meaning (p. 12) as well as emotion and body responses. As shown in Figure 3, after an initial pre-classification (most presumably regarding situational context), the model proposes five stages (Leder et al., 2004), occurring in sequence: (1) “perceptual analysis,” where an object is initially subjected to analysis of low-level visual features (e.g., shape, contrast); followed by (2) “implicit memory integration,” in which art is processed via previous experiences, expertise, and particular schema held by the viewer. This is followed by (3) an “explicit classification,” where one attunes to conceptual or formal/artistic factors, such as content and style, and (4) “cognitive mastering,” in which one creates and/or discovers meaning by making interpretations, associations, and links to existing knowledge. The process ends in (5) a stage of “evaluation,” where processing outcomes combine, culminating in both aesthetic judgment and the potential for “aesthetic emotions.” The model also makes a distinction between “explicit” and “implicit” processing (see timeline), with the first two (or possibly three) stages occurring automatically or with little conscious awareness (Tinio, 2013). In latter stages, there is then a component of self-aware or self-referential processing, where the perceiver “evaluates his affective state and uses this information to stop the processing once a satisfactory state is achieved” (Leder et al., 2004, p. 502).

This model offers a number of advancements from previous work. Because all stages feed into a continuously updated state (Leder et al., 2004), it affords a more holistic understanding of how one comes to evaluations or responses. In addition it incorporates a number of factors—emotion, viewer experience, and formal aspects of artworks—to these stages, which partially influence final results. Thus, the model can be used for both a top-down, mechanism-based evaluation of the general processing of art, or for bottom-up, experience-based testing of hypotheses for specific sequences that may inform particular varieties of response (Vartanian and Nadal, 2007). Because of its emphasis on fundamental cognitive mechanisms, the model has also been used in a number of areas outside art—e.g., design, dance, and music (Leder, 2013 for review).

#### Outputs

Primarily, this model proposes two outputs—aesthetic **Appraisal** and **Affect**. These are mainly explained as a result of successful visual/cognitive processing. The authors claim that part of the pleasure derived from looking is the feeling of having grasped the meaning, thus understanding an artwork results in reward-related brain activation. Looking at Figure 3, emotion or assessment come about by moving through each stage, especially “cognitive mastery,” to a successful end. This argument is in keeping with a number of approaches—most notably Berlyne's concept of curiosity/interest and Bartlett's (1932) (1932; see Belke et al., 2010) “effort after meaning”—which stress importance of intellectual engagement or understanding as core dimensions of positive response. The model also notes meaning-making **(Meaning)**, suggesting that this comes through classification and implicit memory integration in which one connects a specific work to an interpretation. Finally, the model accounts for profound or **Aesthetic** experience. This is argued to derive from the natural extrapolation of the cognitive mastery process, whereby the more completely one can master a work, the more harmonious and pleasurable the outcome, occasionally to the extent that one experiences a pleasurable, “flow”-type experience (Csikszentmihalyi, 1990; Leder et al., 2004).

#### Inputs

The model also mentions several inputs (left side, Figure 1). Primarily, the stages of implicit memory integration and explicit classification are argued to be influenced by previous art experience—determining whether one first sees, for example, a “post-impressionist work,” a “sunflower,” or a “Van Gogh” (Belke et al., 2010). Previous experience or expertise also impact assessments of prototypicality and fluency within the second stage—which influence positive/negative emotions and evaluations (Leder et al., 2004, 2005). Explicit classification also involves processing of style and content, driven by personal viewer characteristics such as knowledge and taste (Leder et al., 2005; also Hager et al., 2012) and understanding of art historical context (e.g., see Bullot and Reber, 2013). More recently, an updated discussion of the model in regards to emotion (Leder et al., 2015) suggested that the switch between aesthetic or more pragmatic approaches in “explicit classification” may be driven by a check of one's desires for emotion or mood state. In cognitive mastering, where meaning is extracted, lay persons may also be more likely to draw on self-related interpretations like feelings, personal memories, or experience, while experts may rely more on art-specific style or concepts (Augustin and Leder, 2006; Hager et al., 2012, p. 321). Leder (2013) extends this even to classifying objects as “Art.” He notes that top-down classification before the actual episode, may affect experience by engaging an aesthetic mode, regulating hedonic expectations, and thus modulating intensity of emotion or interest.

#### Additions

In regards to the actual outcome of viewing art, the exact relation of specific emotions or evaluations to certain given inputs largely remains unclear. As also noted by Leder (2013), there is need of a more integrated explanation of how emotion or other physiological responses might tie to processing experience. While the model notes the role of personality and experience as a driver of outcomes, and the specific stages where self components may have an impact, it does not consider how these aspects are actually integrated or acted upon within psychological experience (see also Silvia below)^7^. There is also need for more explanation of how art-viewing can alter perceptions or understanding within experience (Novelty, Transcendence, Self Adjustment). While acknowledging potential for such results, it remains unclear what must happen within specific encounters for a change of the next viewing moment or the next experience. Presumably, the process might follow our additions (far right: Figure 3), where specific mastery in one encounter (or even within one stage), mediated by positive feedback or emotion/evaluation, would allow one to add to memories/experiences, which would then modify the self. A related output might also be posited for art's longitudinal impacts (Social, Health).

Another issue involves disruptions. Recent work by Leder's group (Jakesch and Leder, 2009) has shown the importance of ambiguity or breakdowns in the mastery process. This is displayed in the “evaluation” stage, and may create a more intense experience by causing one to undergo another loop of the model. However, the specifics of how these arise and create intense vs., for example, negative reactions could be more fully addressed. As it stands, the model appears to afford only a one-way mechanism for improving mastery, which leads to pleasurable experience. This raises the question of how one overcomes difficulty or finds new interpretations (Leder, 2013). The model has also not been connected to specific negative outcomes or physical action. These could presumably be placed as one more component of affective state (bottom).

### The late stages: Silvia et al.: appraisal theory and emotion with art

Several approaches also expand past the models above to consider in more detail later processing elements. First, the work of Silvia (e.g., Silvia, 2005a,b) specifically builds on the ideas of Leder, emphasizing information processing and visual art. However, Silvia's approach focuses on the mechanisms for arriving at specific emotions and artwork assessments, while it simultaneously questions previous psychobiological, prototypicality, and processing fluency approaches.

Silvia (Silvia and Brown, 2007) argues that past processing theories had two major limitations: First, their use of fluency or typicality as a main determinate of positive or negative reactions leads to difficulties in explaining why specific emotions would arise beyond this basic affect. Specifically, previous theories could not discriminate between emotions. At best, they proposed an undifferentiated feeling of aversion or interest. Second, reactions to works could be both positive or negative, but this would depend on other contextual factors such as personality rather than just ability to fluently process. Further, it is especially “hard to derive” what feelings arise from not-fluent or non-prototypical interactions with art (Reber et al., 2004). In response, Silvia proposed an approach based in appraisal theory (e.g., Scherer et al., 2006) that connects reactions with the personal relationship between viewer and art. He argues that each emotion has a distinct appraisal structure or set of evaluations that evoke the response. These evaluations are inherently contextual and subjective, with the central assumption being that evaluations, not the object, are the local cause of experience.

**Figure 4:**
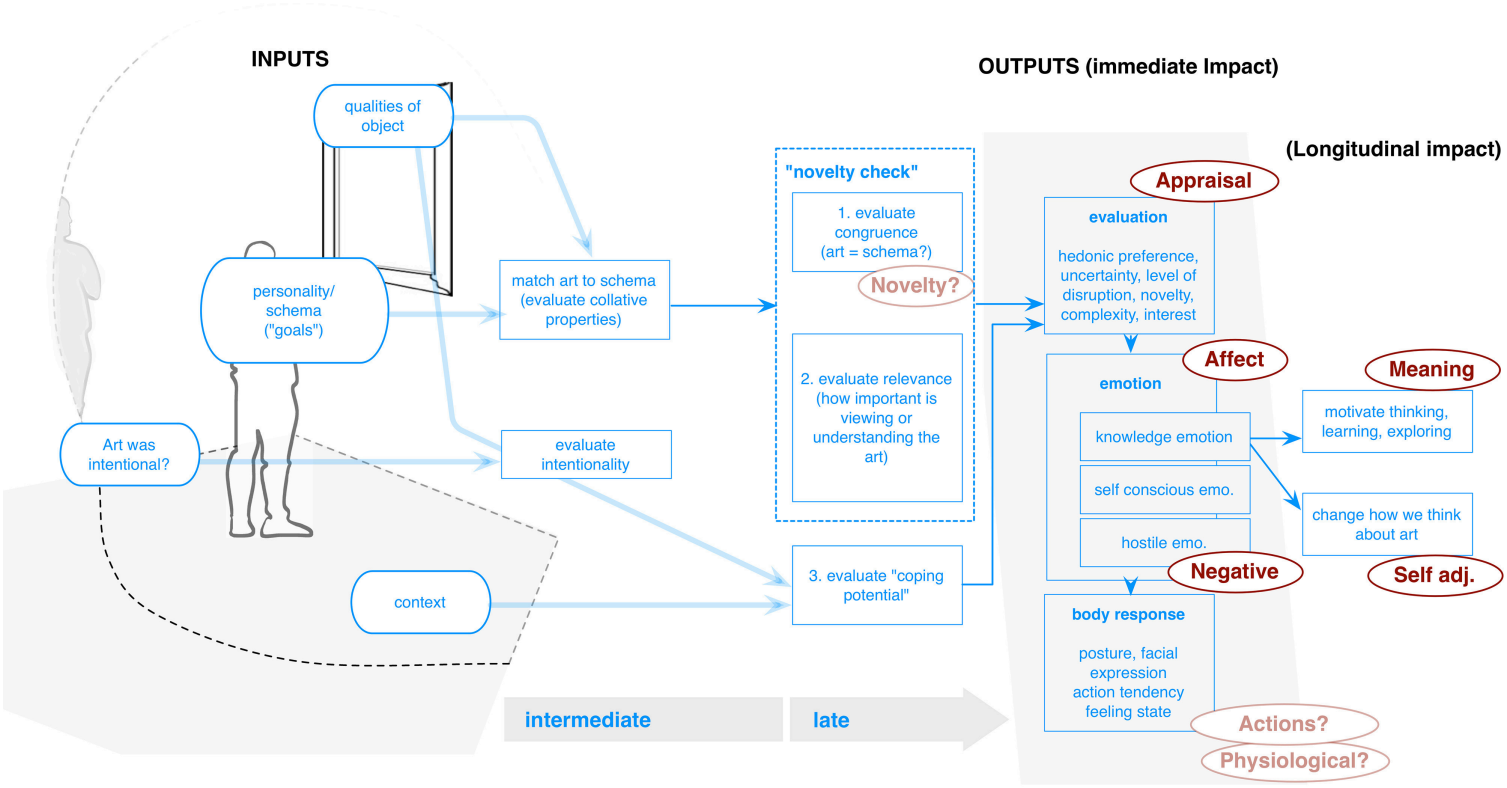
**Silvia model (created by the authors for this paper)**.

As shown in Figure 4, where we have produced a model based on his arguments, Silvia proposes that responses can be broken into two main components: (1) There is a “novelty check” (Silvia, 2005a, p. 122), which is connected to processing of “collative” factors (following Berlyne)—referring to the relative understandability, interestingness or uniqueness of art. This is also tied to the matching of object to the existing schema or expectations of the viewer, and might be further divided into both basic “congruence” and the relevance that the object or the act of matching has to one's goals or self (Silvia and Brown, 2007), with the output being a feeling of relative ease and understanding. (2) There is also “coping potential,” or estimate of relative control or efficacy within the situation itself (Silvia, 2005a). Throughout his writing, he also includes a third factor, relating to (3) relative importance of the object/situation to the self.

For example, in the case of interest (Silvia, 2005a, 2006), the appraisal structure would consist of: (1) a judgment of high novelty/complexity (i.e., low schema congruence), combined with (2) high coping potential, and (3) low self relevance or little importance for one's goals or expectations and thus low perceived threat. In contrast, anger (Cooper and Silvia, 2009) would combine appraising an event as (1) inconsistent with one's schema (low schema congruence), but also (2) with low coping potential (e.g., as action beyond one's control) and (3) closely tied to one's goals/self. Because of this structure, Silvia concludes that in any situation, different people will have different responses to these processing checks, and thus different emotions to the same stimulus, or the same person may even have different emotions depending on context (Silvia, 2005a). Like Leder, Silvia also emphasizes that responses need not require overt awareness.

#### Outputs

**Figure 5:**
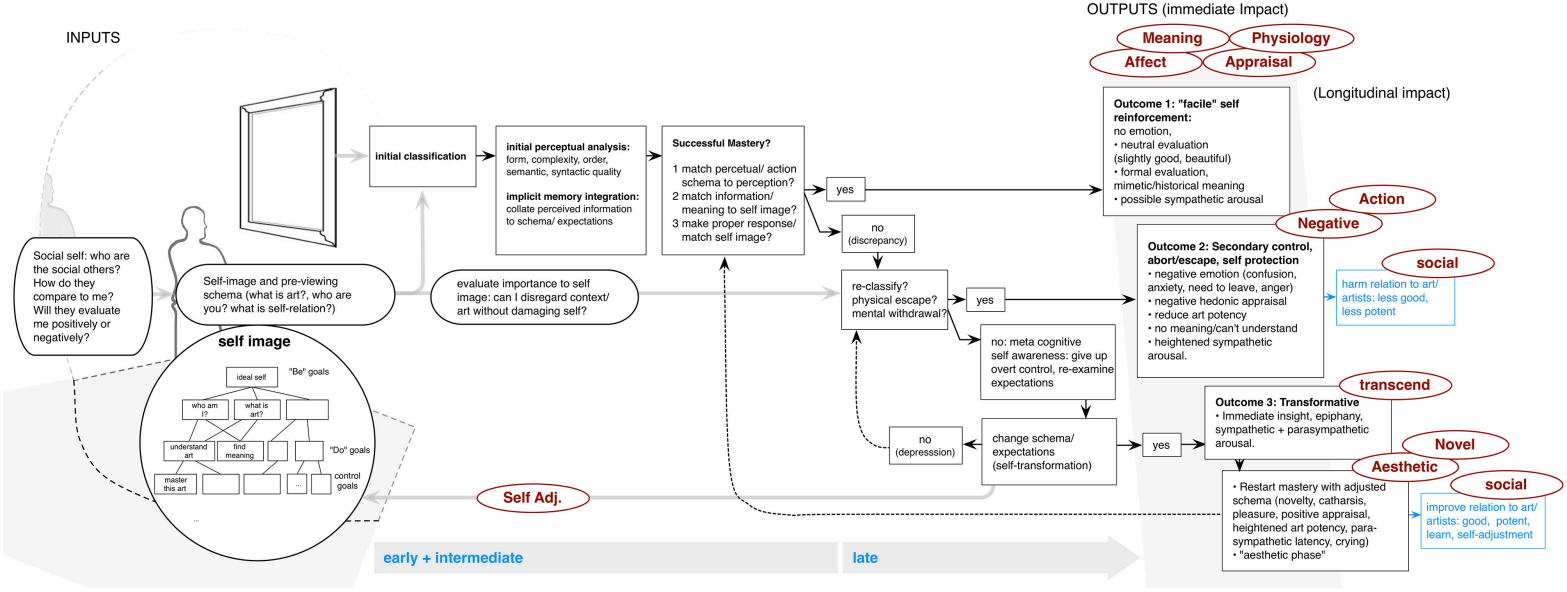
**Pelowski model (adapted from Pelowski and Akiba, 2011)**.

This model also makes an important contribution, especially regarding art impact. The two main outcomes, as in the above models, are **Affect** and **Appraisal**. Silvia however adds to the previous models by proposing pathways for specific reactions (Silvia and Warburton, 2006), giving a frame for empirical assessment of experience. By assessing the processing checks noted above, typically via Likert-type assessments,^8^ the model can explain why people have different emotions to the same event, and why different personality traits, skills, and values can predict responses (Silvia and Brown, 2007). This also enables movement beyond simple pleasure and preference, to surprise, confusion (Silvia and Nusbaum, 2011) as well as **Negative** responses such as anger, disgust, contempt (Silvia, 2009). As shown in Figure 5, these are specifically explained as arising from low congruency and differences in relative coping and self relevance. Beyond emotion, Silvia also groups outputs into clusters (Cooper and Silvia, 2009, p. 111), noting that appraisal theories “are componential theories,” which include facial, vocal, postural expressions or other **Actions**, as well as **Physiological** response.

The model also posits categories of responses that specifically tie to different self-art relations. He notes “knowledge emotions” (interest, confusion, surprise), which are tied to intellectual matching of stimuli to schema, typically in high coping contexts. “Hostile emotions” (anger, disgust, contempt), hinge on threat to goals/self. “Self-conscious” emotions (pride, shame, embarrassment) tie to appraising events as congruent or incongruent with one's goals, and are also viewed as being caused by oneself rather than external events (Silvia, 2009). In the knowledge emotion category, Silvia (2009, p. 49) also notes the role of meta-cognitive reflection on the self. These emotions “stem from people's appraisals of what they know, what they expect to happen, and what they think they can learn and understand.” With self-conscious emotions, “to experience feelings like pride, shame, guilt, regret…[we] must have a sense of self and the ability to reflect upon what the self has done” (p. 50). In both cases this reflection might act as a conduit to **Adjustment**/learning and/or creation of **Meaning**. Especially knowledge emotions may “motivate learning, thinking, and exploring, actions that foster the growth of knowledge” (Silvia, 2006, p. 140), and would come about as an output of one model cycle. It is also presumably through the earlier matching of schema to art—or rather in mismatches, paired with acceptable levels of coping/relevance—that one encounters **Novelty** or changed perception.

In the discussion of hostile emotional responses, the role of the self relates to attack on identity or schema. Responses stem from a “deliberate trespass” (Silvia, 2009, p. 49) against one's goals/values. These responses are tied to action that is often given as a means of maintaining or protecting the self, motivating aggression and self-assertion (Silvia, 2009). Finally, he notes potential longitudinal impact (**Self Adj**.), claiming “a consistent finding …is that training in art affects people's emotional responses…[and] changes people's emotional responses by changing how they think about art” (Silvia, 2006, p. 140). He links this especially to knowledge emotions experienced in the art processing experience.

#### Inputs

Silvia also explicitly connects responses with inputs, specifically personality. He notes that perhaps the most important aspect is how events relate to important goals or values (Silvia and Brown, 2007). This was empirically considered, for example, in his finding of changes in correlation between complexity/coping and interest depending on other individual personality factors (Silvia, 2005a). Elsewhere, in a study on chills and absorption, Silvia found that people high in “openness to experience” as well as art expertise reported more such responses (Silvia and Nusbaum, 2011, p. 208).

#### Adjustments

Questions remain, especially regarding the ordering of the three assessment checks. Specifically, while previous writing tends to present them with only a rough order, one could argue that this would most likely not be the case. One might question whether there is a primacy of assessment for either schema congruence or self relevance. It could be argued that with low relevance, the outcome of the congruence check has little meaning. On the other hand, individuals may be predisposed to constant checks of congruence, relating to basic processing or self-preservation, and thus this assessment may often come first. Another question regards when and how we reflect on our experience. While Silvia does explain reactions in terms of self-related assessments, he does not detail exactly what kind of mechanism this would require. This is most clear in discussion of hostile reactions. Arguing against prior fluency or prototypicality approaches, Cooper and Silvia (2009, p. 113) claim “it seems unlikely” that people have hostile reactions to art “because they find it insufficiently pleasing, prototypical, meaningful.” Instead, “people appraise art in ways that evoke hostile emotions, …in short, some art makes some people mad.” However, this argument is rather circular. Therefore, it would be useful to go one step further, and visually articulate—within a model—*why* this might be so at the individual level. Similar discussions could also be made of specific actions or body responses. His approach also does not divide between automatic and more reflective experience. This question, he notes is “an intriguing, cutting-edge area of appraisal research” (Silvia, 2005b, p. 6). While noting that we might be changed from our experience and that emotional responses may change throughout art exposure, there is no explanation of how this might develop within experience itself (Silvia, 2006).

### Pelowski et al.: discrepant and transformative reactions to art

The model of Pelowski (Pelowski and Akiba, 2009, 2011) was also conceived as an extension of the Leder approach, with the addition of some appraisal theory elements, and attempts to refocus on the specific discussion of changes or evolutions in responses within the art interaction experience.

Pelowski et al. argue against a typical emphasis on harmony, fluency, or immediate understanding, and instead advocate a more labored process of discrepancy and subsequent adjustment. Pelowski and Akiba (2011) note that many past discussions of art experience—both theoretical and anecdotal—involve some means of disruption or break from the flow of everyday life experience. It is these untypical reactions that are argued to actually constitute the impact and importance of art, acting to disrupt a viewer's pre-expectations and forcing upon them a new means of perception or insight (see also Pelowski et al., 2012; Muth et al., 2015 for similar argument). Yet, these very qualities are often eliminated from the study of art perception. They further argue that models have come to “equate art perception to either an emotional/empathic alignment of viewer to artist or artwork” or to a cognitive “assessment of an artwork's …information” via matching of schema to the object of perception, leaving models “without a means of accounting for fundamental change within art experience” (Pelowski and Akiba, 2011, p. 82). Like Silvia, they also argue that current approaches cannot describe how individuals arrive at specific reactions, limiting researchers' ability to unite cognitive, emotional, and evaluative reactions within experience.

Their model (Figure 5) posits five stages, beginning with a specific conception of expectations or viewer identity. Pelowski and Akiba (2011, p. 87) argues that viewers carry “fundamental meanings regarding themselves, other persons, objects, or behaviors—‘Who am I?’ ‘What is art?’ ‘How does art relate to me?”’ which collectively combine to form what they refer to as the “ideal self image.” Updating previous work by Carver (1996), Pelowski and Akiba (2011) specifically posit a theoretical construction for this self, which can be considered as a hierarchical pyramid with core traits (“be goals”) at the top, and branching down to expectations for general actions (“do goals”), and further subdivided into more specific schema. Through connection of low-level schema to core ideas of the self, all action (such as viewing art) then involves application of this structure. This occurs in tandem with human drives to protect the self image, through cognitive filters that lead attention away from potentially damaging information. Thus “success or failure in perception, as well as what individuals can [initially] perceive or understand, are a result of this postulate system” (pp. 85–87), and provide the mechanism for understanding reaction to art.

The authors then propose three main outcomes. First, individuals attempt to successfully match schema to art by classifying and understanding, coinciding with the “cognitive mastery” in the Leder model. They also acknowledge that this outcome is a general goal of most experience, and may induce pleasure, harmony, or even flow-type states. However, because this outcome marks a matching of schema to perception, mastery also would coincide with a “facile” reaction to art, reinforcing previous expectations and cutting off possibility for new perception or insight. Moving past this point, they argue, requires some “discrepancy” within experience. This can involve any number of aspects—e.g., between expectations for perception and art, between meaning and prior concepts, between bodily reactions and expectations for how one should act. In each case discrepancy acts to “bump” an individual out of their preconceived frame (Pelowski et al., 2014, p. 4), forcing response or adjustment.

Upon discrepancy, the model then posits that individuals move to a “secondary control” stage in which they try to diminish or escape from the discrepant element. This is accompanied by actions—e.g., re-classifying art as bad or meaningless, diminishing importance of the encounter, or physically moving away—which avoid a questioning of higher-order aspects of the self image, and also explain the negative emotional or evaluative experiences sometimes had with art. On the other hand, if viewers persist, they may instead eventually alter their own schema in order to better approach the art. This is argued to be most likely when art-viewing has a fundamental tie to the self (representing a higher order goal) and one cannot easily escape (Pelowski and Akiba, 2011). This change also coincides with a shift from direct perception to a more meta-cognitive perspective, in which viewers give up previous attempts at control, acknowledge discrepancy, and eventually create new schema for viewing the art. They conclude that it is this process whereby one “transforms” the self, that can be connected to change, novelty, or insight, and coincides with highly positive emotion, and deepened or harmonious engagement.

#### Outputs

This model is especially important for explaining both highly positive (**Aesthetic**), and **Negative**, as well as insightful or **Novel** and **Transcendent** reactions. The model also unites these within one progressive experience. It is argued that in order to arrive at the final outcome a viewer moves through all antecedent stages (Pelowski et al., 2012). In this vein, one of the model's key benefits is its division into specific stages, tied to application, protection or adjustment of the self. Thus the authors can attach general theory regarding various reactions noted for each of these events (see also Leder, 2013). Notably, they suggest emotion or **Affect**, which, following Silvia, would arise in specific clusters depending on the positive or negative experience of applying the self. They argue that empirical analysis of viewing art would be expected to show a progression from no emotion in the facile stage, to confusion, anxiety, and tension, followed by anger in secondary control, and finally self-awareness, epiphany, or happiness in the aesthetic stage. This division has also been supported by recent empirical evidence (Pelowski et al., 2012; Pelowski, 2015). Similar results are also tied to **Physiology**, specifically heart rate and skin conductance. They argue that the first stage should show little physiological response, while secondary control would lead to sympathetic (fight or flight), schema change to both parasympathetic and sympathetic, and the final stage to parasympathetic return to homeostasis. This final outcome was also recently tied to crying (Pelowski, 2015). They also suggest specific **Action** (need to leave) in the “secondary control” stage.

Regarding **Appraisal**, they also argue that evaluation ending in cognitive mastery may reveal appraisals that aid in ignoring or assimilating discrepancy (Pelowski and Akiba, 2011). On the other hand, in secondary control, self-protectionary strategies would manifest in negative hedonic appraisals, as well as lower “potency or activity” (e.g., Osgood et al., 1957). They further consider **Meaning**, dividing outcomes into three modes: (1) initial assessment for surface or mimetic qualities in cognitive mastery, (2) meaninglessness in secondary control, and (3) “a fundamental change in viewer-stimuli relation” in the last stages, where art meaning is tied to metacognitive reflection on its impact and the preceding psychological experience (Pelowski et al., 2012, p. 249).

The model is also particularly unique in its explanation of changes or transformation with art. Recently, Pelowski et al. (2014) connected this outcome to goals of museum/art education, and pointed out its equivalence to discussions of insight or creativity. By breaking from the mastery process through the introduction of meta-cognitive assessment and schema-change, followed by re-engaging in final mastery with a new set of schema, they argue, “we introduce a means of explaining the transcendental quality of art” and of “connecting the existing conception of mastery to …novelty and personal growth” (Pelowski and Akiba, 2011, p. 90). They also posit longitudinal impact, tied to changes in schema or the “hierarchical self.” Especially the aesthetic outcome may manifest in re-evaluation of a viewer's own self image, which may be detectible in paired self-evaluations before and after viewing (**Self Adj**.). They also argue that both the abortive or transformative outcome may cause change in individual's relationship with the class of art or artists, involving hedonic and potency evaluations (**Social**), and may spur individuals to seek out/avoid other encounters.

#### Inputs

Regarding inputs, the model considers the role of specific expectations or personality, and goes further to place these within a theory of the self. The authors note that “those who have a strong relationship to a stimulus, or high expectations for success…are [more] likely to find themselves in the intractable position” leading to aesthetic experience (Pelowski et al., 2012, pp. 246–247). This might be tied to training in the arts, or might affect those who identify as art lovers, who have a high need to find meaning in artworks, or who have the general need for control. More recently, the authors have also taken into account the physical and social situation, noting that the environment, especially when one is among others who one considers more knowledgeable, may be likely to evoke facile or negative experience, tying to a need for protecting the self (Pelowski et al., 2014).

#### Additions

At the same time, the model has a conceptual focus, laying emphasis on schema and overlooking much of the way individuals might often respond to art (basic perceptions, mimetic evaluations, or pre-reflective experience). A recent review (Leder, 2013) also noted that it is “more descriptive than formalized,” requiring transformation into “more operative versions with process-based rules,” quantifying what feature of a representation at one stage affects latter stages.

### Cupchik: detached/aesthetic and pragmatic approaches to art

**Figure 6:**
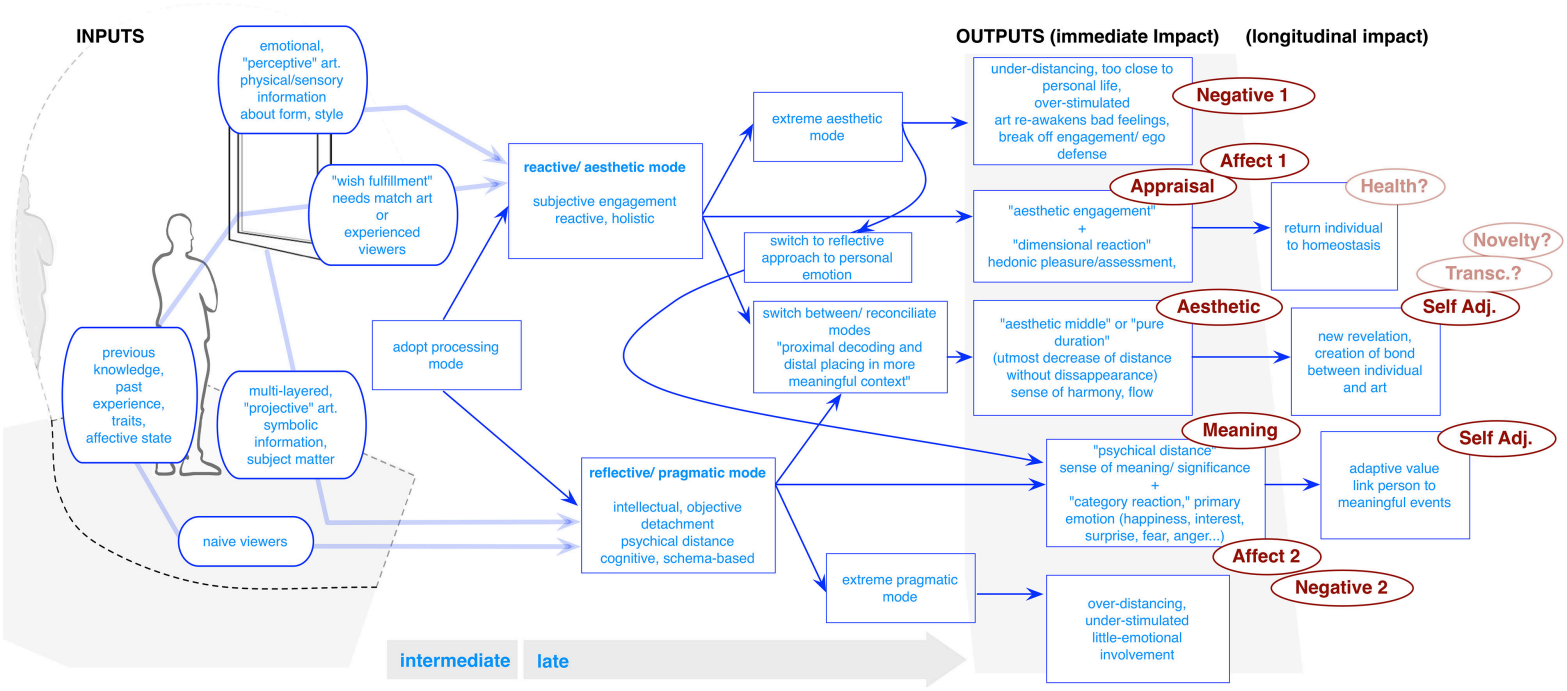
**Cupchik model (created by the authors for this paper)**.

Finally, the theories of Cupchik have also not yet been placed into a unified model, but have individually been instrumental in empirical art research. These also involve several themes which can be connected to form an understanding of art experience,^9^ and thus were deemed an ideal target for this paper (Figure 6).

Similar to other cognitive approaches, Cupchik views art experience as a meeting of object, environment, and personality factors (Cupchik and Gignac, 2007). In order to understand their interaction, a main theme regards two modes of responses (Cupchik, 2011, p. 321): “everyday pragmatic” and “aesthetic.” The way that these modes are integrated, and often which of the two the viewer employs, determine the outcomes of experience. The pragmatic involves a predominantly cognitive, schema-based assessment, in which one assesses meaning and significance. The “aesthetic,” on the other hand, involves integrating context, memory, and physical/sensory qualities “associated with style and symbolic information” (p. 321). This involves a more reactive or “holistic” appraisal “in which specific codes for interpreting are bound with affective responses that map onto dimensions of pleasure or arousal.” Cupchik (2006, 2013) also posits an alternative naming for this division, suggesting a contrast between “subjective engagement” and “objective detachment.” While subjective engagement is based on intense personal responses, objective detachment reflects a more intellectual treatment^10^.

In engaging art, one may switch between modes in response to different information or one's processing experience. Cupchik (2011, 2013) notes that the two modes' “extreme conditions” can also lead to unwanted experience. This would involve either “underdistancing,” in which subject matter reminds us of troubling aspects of our personal lives, or causes unwanted emotion, or “overdistancing,” when design aspects push one too far away (as in some avant-garde art), with little emotional involvement (Kemp and Cupchik, 2007). The most pleasant responses may occur when individuals find an “aesthetic middle” (Fechner, 1978) between absorption and detachment—“utmost decrease of distance without its disappearance” (Cupchik, 2006, p. 217). Cupchik (2013) also argued that one appeal of art is the opportunity at reconciliation between modes. While he argues that it is not possible to be in both modes at the same time, we can “shift rapidly between the two” (2011, p. 321). Thus, it is “the capacity of a work of art to be grasped, elaborated, and experienced in several systems” that makes it compelling (p. 294).

#### Outputs

The model can further be articulated through its discussion of outputs. First is its discussion of **Appraisal**, which it connects to the aesthetic or reactive mode. Cupchik notes potential for **Meaning making**, via reflective and/or pragmatic processing. **Affect** is also noted. In an earlier publication, Cupchik (1993, p. 179) suggested that two kinds of emotional processes might in fact be distinguished, which would coincide with either the analytical/schema-based or holistic/experiential processing modes. These can be termed “dimensional” and “category” reactions. The former are closely tied to bodily states of pleasure/arousal caused by a particular stimulus. The latter pertain “to primary emotions,” such as happiness, interest, surprise, fear, anger, and emphasizing spontaneity or empathic reaction to art. More recently, Cupchik (2011, p. 321) suggested that the former affect type may accompany aesthetic experiences “from the first moment of perception,” giving as evidence studies involving displaying artworks of differing complexities or affective contents for very short durations, and where participants would avoid a second viewing based on their ability to detect lack of order or unwanted emotional valence^11^. In turn, the primary emotions are associated with longer exposure durations “that enable a person to situate the work in the context of life experiences” (Cupchik and Gignac, 2007). He also notes that this often touches the reflective mode of appraising, which is more closely related to emotions linked to the self (Cupchik, 2011). He suggests that both cases may lead to **Negative** affect. This would come from either: (1) difficulty in initially processing art, leading to hedonic aversion through the reactive/aesthetic mode, (2) “under-distancing,” where the work is too close to one's self and/or a troubling situation, or (3) negatively perceived content in art as processed in the reflective mode.

Cupchik (2011, p. 321) also argues for adaptive impact from emotional art response (**Self Adj**.), noting especially “primary emotions” have adaptive value, “because they link the person to meaningful situations.” In turn, the reflective mode (Cupchik, 2011, p. 321) can be related to the principle of “emotional elaboration,” which implies that a person searches for underlying layers of meaning, in part due to the prompting from their affective experience, and might be connected to growth/self-adjustment (Kemp and Cupchik, 2007). Especially when individuals are able to find the proper emotional distance, they may enter a state in which they break from a “normal outlook” and achieve new points of view or approaches (Cupchik, 2006, p. 216). He also suggests that this can lead to a state of consciousness involving “suspension in the experience of time—a frozen moment in which the person and the work become one” (Cupchik, 2013, p. 85). This event then might be connected to **Aesthetic** experiences, as well as **Novelty** and **Transformation**. His idea of aesthetic engagement has the posited impact of returning an individual to homeostasis or harmonious interaction with the environment, which might serve as an avenue to longitudinal impact (**Health**).

At the same time, Cupchik and Wroblewski-Raya (1998, p. 65) note that while art can be used for wish fulfillment or revelation, it can also evoke “ego-defense.” “The subject matter …might also resonate with unresolved issues and needs.” This might lead to a “defensive intellectualizing response” in which the individual escapes or avoids processing, by, for example, focusing on its style or other benign elements. This could also lead to an adaptive moment. “The artwork …mirrors the person's life and externalizes what has been a private concern,” thereby providing “tension release.” This may involve ability to adopt some distance, which “permits the person to experience the emotion without having to address its consequences.”^12^ This too might connect to a change in perception (**Novelty**) or insight, while the former outcome may lead to **Negative** emotion/responses. Cupchik (2006) also notes that while one can of course employ a pragmatic approach, in order to appreciate art as “Art,” one *must* shift to an aesthetic frame.

#### Inputs

Regarding inputs, Cupchik primarily notes personality. For example, in an empirical study (see Cupchik and Gignac, 2007) he showed the impact of previous experience when determining what aspects a viewer might attend to, concluding that art-naive viewers generally focus on subject matter because it is easier to discern than style. The latter is more likely to be attended to by experts, which he connects with a reflective mode and desire for challenge^13^. On the other hand, much like Leder, he discusses the importance of previous experience when adopting an “aesthetic” mode. Individuals “bring appropriate codes of interpretation and engagement. [One takes] this for granted until encountering a new form of artistic expression,” which individuals do not know how to respond to (Cupchik, 2013, p. 73). Cupchik (2006, p. 214) also notes the present and/or desired affective state may play a role in art selection and attention. “People can intentionally modulate their states of pleasure or arousal by selecting stimuli that possess a needed quality.” This “wish fulfillment” (Cupchik and Wroblewski-Raya, 1998, p. 65) might itself be largely involved when we take a “reactive” mode of appraising art, allowing return to homeostasis (Cupchik, 2011). Finally, Cupchik (2013, p. 85) notes the implicit role of personality in discussion of more profound or harmonious experiences. He suggests that proper distancing may come through cases where “a work expressively embodies a person's sense of identity.” One may alter the mode of appraisal to escape from implications raised by art. He showed this by confronting viewers, who identified as lonely, with paintings depicting lonely scenes, and who were more likely to focus on style than content (Cupchik and Wroblewski-Raya, 1998).

#### Additions

Unclear aspects regard the two modes of appraisal. Much of Cupchik's discussion implies that these modes might occur roughly in parallel, or that individuals can actively select which mode to employ. However, his research on short and long time sequences seems to imply that we immediately take a reactive approach to assess the basic object. This would seem to fit to the work of Leder, Chatterjee etc. This raises the question of whether one mode might influence the other or how this might occur. Even more, his discussion of under-distancing and its ability to lead to ego defense or learning and growth, might imply a switch between an initial reactive to a reflective mode, at a late stage of experience. It may be useful to parse what would be these outcomes' differences.

## Discussion and conclusion

This paper had the goal of taking existing theoretical explanations of the psychological processing of art, and placing these into a unified visual basis for the purpose of articulating how, *and if*, they address specific outcomes from our art experience. These outcomes were also tied to inputs or contextual factors, and general processing stages. Through this review, we hoped to both provide a new tool for discussing the modeling of art, displaying how models may differ or overlap, and providing a more general window into the present state of art psychology research. We conclude with a short discussion of these models' synthesis, and suggestions or implications for future research.

### The state of art modeling: some agreement on outputs; many paths for how they are achieved; many avenues for empirical investigation

First, concerning outputs or psychological implications, as noted in the introduction, these factors might be said to drive art's psychological interest, and are thus the prime targets for modeling itself. This was also a main contribution of our paper, which sought to identify a range of potential outputs and label these when they were considered in the specific models. For the purpose of quick comparison, all outputs are summarized in Table 1, which denotes whether or not they are explicitly or implicitly included in each of the reviewed models, or omitted. This table also provides a similar review for inputs. For more extensive comparison, we have also provided a brief synopsis of each model's specific explanations for outputs in Table 2 **(Parts 1 and 2)**.

**Table 1:** **Overview of explicitly mentioned inputs and outputs in models of art experience**.

	**Chatterjee**	**Locher et al.**	**Leder et al.**	**Silvia et al.**	**Pelowski**	**Cupchik**
**INPUTS**						
Personality	–	○	–	○	○	○
Prior affective state	–	○	○	**–**	–	○
Memory, knowledge	○	○	○	○	○	○
Art display, context	–	○	○	•	○	–
Artwork qualities	○	○	•	•	–	○
Social/cultural setting	–	○	○	•	○	–
**OUTPUTS**						
**Basic affect/body**						
Affect	○	•	○	○	○	○
Physiology	•	–	–	○	○	–
Action	•	○	–	•	○	–
**Info. processing**						
Appraisal	○	○	○	○	○	○
Meaning	○	○	○	○	○	○
Novelty	•	•	•	•	○	•
**Art-specific reactions**						
Transcendence	–	–	•	–	○	•
Aesthetic/detached	•	–	○	–	○	○
Negative	•	–	–	○	○	○
**Longitudinal impact**						
Self Adjustment	•	–	•	○	○	○
Social	○	–	•	–	○	–
Health	–	–	•	–	–	•

*Circle (○) signifies explicit mention and discussion of Input/Output factor by author(s). Dot (•) signifies implicit mention. Dash (–) signifies no mention*.

**Table 2:** **Models of art experience and noted Outputs**.

**PART 1**						
**Model**	**Basic affect/body**			**Information processing perception**		
	**Affect (positive emotion)**	**Physiology (heart rate, skin conductance)**	**Action (physical/eye movement, gesture)**	**Appraisal**	**Meaning (meaning-making)**	**Novelty (change/adjustment in perception)**
**Chatterjee** Early + Intermediate processing	Primarily tied to visual processing experience and successful classification, identification and understanding.	(?) Aesthetic experiences can enhance cortical sensory processing.	(?) Aesthetic experiences can enhance cortical sensory processing and thus eye movement.	Primarily tied to visual processing experience and successful classification, identification and understanding.	Result of processing of objects, extraction of prototypes, connection to memory, and final decision.	(?) Aesthetic experiences can enhance cortical sensory processing and thus new attention.
**Locher et al**. Early + Intermediate processing	(?) Outcome of visual processing and integration of information with memory.		(Eye movement). Driven by initial pre-conscious processing for gist followed by detailed assessment and influenced by object qualities and viewer personality/expertise.	Outcome of visual processing and integration of information with memory.	Outcome of visual processing and integration of information with memory.	(?) Possible outcome of augmented information and feedback from art processing to memory/personal context.
**Leder et al**. Early, Intermediate, Late processing	Primarily tied to intellectual/processing experience and successful mastery or understanding.			Primarily tied to intellectual/processing experience and successful mastery or understanding.	Through classification and implicit memory integration.	(?) May result from feedback at evaluation stage.
**Silvia et al**. Intermediate, Late processing	From combination of processing for collative properties from matching of art and schema (and resulting assessment for goal congruence and relevance) and coping potential.	(?) From combination of processing for goal congruence and relevance) and coping potential.	(?) Action tendencies (fight/flight, avoidance): from combination of processing for goal congruence and relevance) and coping potential.	From combination of processing for collative properties from matching of art and schema (and resulting assessment for goal congruence and relevance) and coping potential.	Primarily tied to intellectual matching of stimuli to schema, typically in high coping contexts, with resulting reflection and motivated by “knowledge emotion.”	(?) Presumably tied to mismatches between schema and art (low congruency) with sufficient coping and goal relevance.
**Pelowski** Intermediate, Late processing	Determined by relative stage and type of self-engagement/self protection. Classed into three main outcomes: little emotion, negative emotion in secondary control, highly positive.	Determined by stage and type of self-engagement/self protection. Classed into three outcomes: little response, sympathetic fight/flight reaction in secondary control, parasympathetic response in aesthetic phase.	Need to leave, fidget, clap, talk may be tied to self protection strategies in Abortive outcome (Secondary control).	Determined by relative stage and type of self-engagement/self protection. Classed into three main outcomes: facile, negative, highly positive.	Brought about by creation of new schema (self image) via previous process of facing and overcoming discrepancy. Allows one to reset engagement with new schema allowing novel ideas/concepts.	Brought about by creation of new schema (self image) via previous process of facing and overcoming discrepancy. Allows one to reset engagement with new schema allowing novel perception.
**Cupchik** Early, Intermediate, Late processing	Result of: (1) analytical/ schema-based processing of content, leading to primary “category” type emotions (happy, sad…) and (2) holistic/experiential processing leading to “dimension” type emotions relating to hedonic affect.			Result of reactive/aesthetic mode. Based initially on assessed complexity or ease of processing.	Result of reflective/pragmatic mode. Based on later integration of context, viewer and work.	(?) Potentially result of: (1) sudden new view of things/revelation via “aesthetic middle.” (2) Adaptation via reflection on personally-related emotion.
**PART 2**						
	**Art-specific/highly notable reactions**			**Longitudinal/contextual impact**		
	**Transcendence (epiphany, feeling of transcendence)**	**Aesthetic “Aesthetic” contemplation (detached, harmony)**	**Negative (negative affect/emotion)**	**Self Adjustment (changed self, relation to art, growth)**	**Social (socio-cultural adjustment)**	**Health (overall wellness, reduced stress etc.)**
**Chatterjee**		(?) Intermediate processing of compelling or pleasing qualities (symmetry, balance, content) may engage frontal-parietal attention circuits, which may lead to “a feed forward system,” in which object attributes engage attention, and attention enhances processing, leading to heightened engagement/pleasure.	(?) Primarily tied to unsuccessful visual processing experience.	(?) Result of aesthetic experience brought about by making special.	Result of aesthetic experience brought about by making special, causing social cohesion.	
**Locher et al**.						
**Leder et al**.	(?) May result from feedback at evaluation stage.	Derived from highly successful mastery experience.		(?) May result from feedback at evaluation stage.	(?) May result from feedback at evaluation stage.	(?) Possibly outcome of positive mastery experience.
**Silvia et al**.			From low congruency plus a felt “deliberate trespass” (Silvia, 2009) against goals and values (low coping).	Primarily tied to intellectual matching of stimuli to schema, typically in high coping contexts, with resulting reflection and motivated by “knowledge emotion.”		
**Pelowski**	Brought about by creation of new schema (self image) via previous process of facing and overcoming discrepancy. Coincides with final “aesthetic phase” of latency following change.	Brought about by creation of new schema (self image) via previous process of facing and overcoming discrepancy. Coincides with final “aesthetic phase” of latency following change.	Result of self-protectionary actions in secondary control stage. Negative emotions/evaluations are used to minimize danger to expectations/self.	Brought about by creation of new schema (self image) via previous process of facing and overcoming discrepancy. May cause positive adjustment with specific work and general class of art.	Relation between self and art or artists may be changed depending on abortive (Negative change) or transformative (positive) outcome. May involve hedonic and potency assessments. Transformative outcome may cause new art interest.	
**Cupchik**	(?) Potentially result of: (1) sudden new view of things/revelation via “aesthetic middle.” (2) Adaptation via reflection on personally-related emotion.	When a work “expressively embodies a person's sense of identity” leads to suspension of perception of time “in which the person and the work become one.”	Based on: (1) difficulty in understanding or initially processing, leading to hedonic aversion through reactive/aesthetic mode, (2) “under-distancing,” where art is to close to one's self, or (3) negative emotional content as processed in the reflective mode.	Result of: (1) primary emotions experienced through reflective mode and “emotional elaboration” where person searches for underlying layers of meaning. (2) bond created between person/work via “aesthetic middle.”		(?) Potentially result of return to homeostasis as result of successful aesthetic engagement.

Output descriptions based on authors' published models and related publications. Factors preceded by a question mark (?) were not specifically mentioned by the authors, but were proposed by the present paper.

Looking at this comparison, it is interesting to note that all models share some common factors. Notably, almost all authors consider emotion and evaluations as main outputs, and also make an explicit connection to meaning making. This itself may tell us something about current modeling, and the present state of understanding and focus in art research. While this review obviously could not consider all approaches important to art, it does suggest that these common outputs may constitute what investigators feel to be important for defining art interaction. These outputs also mark major factors in present empirical assessment. This probably stems from the present information processing focus. Most models also consider several basic inputs, which might be roughly divided into social, contextual, experiential, and personality-derived elements.

At the same time, the models also differ greatly in their explanations for *how* one arrives at these outputs, and connects these elements to different processing components. For example in the case of appraisal, as Table 2 **Part 1** shows, descriptions range from: an emphasis on visual object identification (Chatterjee), integration of vision with memory (Locher), emphasis on intellectual processing experience and understanding of art (Leder), relative matching of schema and self (Silvia, Pelowski), to taking a pragmatic vs. aesthetic mode (Cupchik). This diversity highlights the presently undetermined nature of current art psychological approaches, and the need for more comprehensive and comparative analyses.

Importantly, this also highlights the potential contribution of this paper, and of visual modeling. As noted above, one of the benefits of a visual model approach is that it forces an author to make an explicit connection between processes and outputs, articulating connections where they might be otherwise obscured in written theory. By placing these same outputs in the visual models, tracing back through their processing descriptions, and comparing between approaches, we may create grounds for future empirical research. We have set up this paper to facilitate this approach. We suggest that the reader might use this review as a means of considering the pathways to the various outputs, and thus the underlying factors and processing sequences. These could then be considered in empirical approaches. This review may also contribute to a better understanding of the theories of these individual researchers.

It should also be noted that this review does not imply that one model is “better” in describing outcomes than others. Rather these models are all presumably describing different aspects of the art processing sequence. This also shows in the models' relative emphasis on different general stages (early, intermediate, and late), which lead to different answers regarding outputs. Future studies might use these different models to consider the differential contribution of the posited sequences for determining their relative impact on output factors.

### Missing elements: physiology, health, negative and profound reactions to art

This comparison also highlights factors that appear to be largely missing in present modeling, and by extension psychological art research. When placed side-by-side, it becomes clear that present approaches largely avoid several outputs. Notably, there is a dearth of discussion of negative factors as well as of novelty, change, or transformation. Beyond the immediate processing components, there are other, long-term outputs that appear under-represented—notably art's role in general well-being or health. As noted by Stevenson-Taylor and Mansell (2012, p. 105), “seldom is a rigorous exploration given to ascertaining the effects of psychological change in the long-term. When and how these changes occur is rarely addressed.” This does certainly seem to be the case here. Longitudinal aspects were not directly mentioned by any author. Similarly, social aspects and socio-cultural adjustments also appear under-represented, with the latter only directly mentioned by Silvia. Similarly under-explored are insight, changed perception, and—somewhat surprisingly—harmonious or aesthetic experience. While several authors theoretically note how this might occur (for example Leder argues that it would involve an act of cognitive mastery approaching perfectly fluent matching of schema to work), it occurs nowhere as a specific model output. This raises the question of how these outcomes might actually have a lasting impact. Only Pelowski and Akiba (2011) specifically note how this might occur.

This general omission of factors as well is quite illuminating, and can be traced into present empirical study, as well as needed targets for future research. It has been recently noted that especially the above negative or transformative factors are often overlooked (Silvia and Brown, 2007; Leder, 2013; Pelowski, 2015), and remain prime candidates for future empirical approaches. As well, there have been calls for assessments of art's health or positive benefits on the viewer (e.g., Cuypers et al., 2012). By extrapolating from these missing outputs, we might say that present models and theoretical discussion appear to be missing a large number of consequences that might define the *importance* of art for society or individuals, and thus why art should, for example, be supported by public resources. Models also appear to omit what might be called “second order outputs” or executive behavior consequences of viewing, such as when anger leads to iconoclasm, vandalism, or violence (c.f. Freedberg, 1989). It is also interesting to note that most of the models do not account for the viewer's body, movement, or physiological responses, which might also be considered (Tschacher et al., 2012). These aspects, we would argue, remain key targets for future modeling, which may then allow for better empirical assessments. Interestingly, as we have tried to show in the suggested additions and updated model figures, many outputs might actually be connected to present model approaches, raising again an avenue for future research and what we hope can be a contribution of this paper.

Regarding inputs, there are also areas for future development. Specific artwork-related aspects such as style are not included in several models (Chatterjee, Silvia, Pelowski). The same can be said for the artwork's historical context, which was also recently argued to be a key processing input (Bullot and Reber, 2013), but in the present review only operationalized as one aspect of the background knowledge of viewers (e.g., by Locher and Leder, but see Pelowski and Akiba, 2011). It also appears that only the models put forward by Leder and Cupchik account for the current psychophysiological and affective state of the viewer. These aspects should be incorporated into the other models and systematically included when setting up experiments. In addition, while most authors specifically note the importance of memory components for processing, and often mention this in their written theory, it is often omitted in the models. This begs for integration and elaboration.

The models' differing discussion of factors, and many of their omissions, are also probably a result of present emphasis on early and intermediate processing stages, and tied to the importance of vision and early neurological components of object recognition. This too suggests a potential fruitful target for future theoretical research. Those models that do focus on late processing (Pelowski, Silvia) are more likely to consider the omitted outputs. This again does not imply that certain models are more or less important: the models that focus on earlier processes may, for example, involve a more detailed consideration of the bottom-up processing of artwork qualities, whereas models with a later focus may concern primarily top-down contributions of the viewer. This speaks to a need for combining these discussions into one processing sequence. Future researchers might consider how the visual processes (e.g., as described by Locher and Chatterjee) feed into the cognitive processes described by Leder, and then lead to the top-down consequences described by the remaining authors. It may also be fruitful to look at the described processing sequences for each output and consider a best solution, given these, and other model's descriptions. While such a synthesis is beyond the aim of the present manuscript, we argue that this is a necessary next step for future research.

### Box and arrow models: limitations and future developments

Finally, a few words should also be given regarding the nature of above models themselves. As noted, they are all box and arrow designs. This represents an important fact in cognitive psychology and discussion of art, because they specifically require theoretical links between inputs, outputs, and processes. At the same time, this method has several general limitations, which future researchers might consider.

It should be clear from this review that while the simple act of connecting inputs to processes to outputs is an important theoretical step for a better understanding of psychological events, the simple arrows that make up many aspects of the above models often do not sufficiently explain how this might actually be accomplished. Many models, especially when visualized, also reveal gaps or confusions in their design. More detail and consideration of individual and contextual factors is often warranted. Many approaches might benefit from more careful consideration of both specific decisions or factors, which can determine specific model sequences, and placement of outputs. While we did attempt to take the step of systematizing the broad components of each approach, we also made the decision to maintain fidelity to the original model interior organization, which in many cases only highlights such suboptimal arrangements. From this review, we would be the first to argue that the field of modeling in aesthetics itself could benefit from more attention to such aspects of visual communication. We hope that future research might consider this.

The linear nature of these models can also lead to a myopic, “false” and often one-dimensional understanding of psychological processes themselves. In reality, these might often occur in concert as complex networks of activation (Cela-Conde et al., 2013), or with individuals cycling back and forth between stages, constantly adjusting and updating expectations, which influences perception and experience. While these aspects were at least addressed in some of the reviewed models (e.g., Pelowski, Silvia, and the discussion by Leder), such complex approaches, require further emphasis, and become even more necessary when taking the next step of connecting sequences to activity in the brain. Further, it may be that future research should even move past the box and arrow design, considering for example novel paradigms such as Bayesian flow models, or predictive processing theory (Clark, 2013, 2015) which posits that the brain operates based on comparisons with automatic predictions of the environment; both result in more complex probabilistic models of outputs or experience.

To conclude, we hope that this review may contribute to such future modeling, and serve as a useful basis for needed future comparative and hypothesis-driven research.

## Author contributions

All authors listed, have made substantial, direct and intellectual contribution to the work, and approved it for publication.

### Conflict of interest statement

The authors declare that the research was conducted in the absence of any commercial or financial relationships that could be construed as a potential conflict of interest.

## Footnotes

^1^For example, for early vision, Chatterjee (2004) argues that occipital cortex and frontal-parietal attentional circuits play the most emphasis.

^2^Noted regions for late vision include: orbitofrontal cortex, insula, temporal pole, ventromedial prefrontal cortex. These are connected to the interaction between cognitive and emotional processes and monitoring of one's own affective state.

^3^This impact is interpreted empirically from the involvement of subcortical components of the reward circuit in aesthetic experiences, notably anterior cingulate cortex.

^4^The model connected emotion to the anterior medial temporal lobe, medial and orbitofrontal cortices, and subcortical structures which mediate emotion and reward systems. Chatterjee (2010) connected appraisal to a widely distributed network, most importantly the dorsolateral frontal and medial frontal cortices.

^5^In bilateral fusiform gyri, angular gyrus, and the superior parietal cortex.

^6^More recently, Locher et al. (2010) integrated this model with consideration of the tactile and sensual aspects of the handling of object and assessment of its use, geared toward discussion of commercial, design products. However, the present discussion will focus only on visual aspects.

^7^Locher et al. (2007, p. 76) also question some of this model's arguments regarding location of processing aspects within its stages. In a study where participants were asked to view paintings and give a running oral report of their processing, Locher et al. noted that some participants' “initial reactions to the artworks would be classified as occurring in stage 4 …involve[ing] deliberate (top-down) self-related interpretations.” Locher et al. argued that their findings suggest that reactions “may occur much more rapidly and automatically than predicted.…suggesting that [individuals] are able to make a rapid evaluation of a picture's content and aesthetic appeal.”

^8^Silvia specifically identified a set of key evaluations for identifying congruency, coping, and self-relevance (preference, uncertainty, level of disruption, novelty, complexity, interest).

^9^Cupchik himself made this acknowledgment and suggestion that his work might be fit into a cohesive model in a recent retrospective address concerning his body of research (2011, p. 320).

^10^See also Cupchik (1995) in which he also uses the terms “reactive” and “reflective” approaches.

^11^He goes on to argue that this “clearly showed that the interaction of cognitive and affective processes in aesthetic perception take place holistically within the first glance” and thus contradicts Zajonc's (e.g., Zajonc, 2000) assumption that preferences are often unmediated by cognitive processing (Cupchik, 2006, p. 212–213). This is a debate for another paper.

^12^Shown empirically by Kemp and Cupchik (2007) who presented viewers in a negative state with a range of positive and negative paintings, and who found that viewers wanted to see the paintings with negative themes a second time, primarily however, “because they evoked thoughts rather than feelings.”

^13^He was specifically referring to literary experiences, however this would presumably also apply to visual art.
